# Evils of Water Drinking

**Published:** 1887-04

**Authors:** 


### EVILS OF WATER DRINKING.

Sir Henry Thompson, the great English teetotaler physician, who refuses to treat any one in illness who drinks alcoholic beverages, says, nevertheless, that the only water which is perfectly safe to drink, unless it has been boiled and filtered, is natural mineral water. In the shape of a wriggling worm, invisible to the eye, even when held to the light, and only to be detected by the microscope, a water drinker may have given more permanent lodging to a snake than ever the hospitable whale gave to Jonah. But an animalcule will grow and thrive in the inside of the indiscriminate water drinker who has swallowed it until it feeds upon his vitals and exhausts his health and strength. The victim wonders why he or she feels so much discomfort in the stomach, loses all appetite for food or else grows ravenous, feels nervous, depressed and incapable of active duty. The unknown and unsuspected reptile stowaway, swallowed weeks or months before, in a glass of impure water, is the cause, and he who doubts the numerous cases on record of inanition, or suspension of life, and apparent death from this cause alone must be incapable of weighing evidence. I have myself known a young girl who died apparently and remained cold and lifeless until put in her coffin, when her mouth opened and a small snake, some few inches long, appeared. A French doctor, who had attended to the case, was, fortunately present, and drew the reptile out with his fingers. After a brief interval, the exhausted girl began to shudder, then opened her eyes and was thus rescued from being buried alive. I heard the story not only from her own lips, but from those of her father and mother, and have not the slightest doubt of its truth. Numbers of similar cases are reported in medical journals. If the mere swallowing of indigestible food will almost deprive a dyspeptic of the use of his limbs and brain, how much more must a living reptile in the stomach paralyze and suspend the functions of the human body.—*Ex.*

